# Comparison of Complication Risk for Open Carpal Tunnel Release: In-office versus Operating Room Settings

**DOI:** 10.1097/GOX.0000000000003685

**Published:** 2021-07-12

**Authors:** Dustin J. Randall, Kate Peacock, Katelin B. Nickel, Margaret Olsen, Andrew R. Tyser, Nikolas H. Kazmers

**Affiliations:** From the * Oakland University William Beaumont School of Medicine, Rochester, Mich.; † Department of Orthopaedics, University of Utah, Salt Lake City, Utah; ‡ Institute of Clinical and Translational Sciences, Center for Administrative Data Research (CADR), Washington University in St. Louis, St. Louis, Mo.

## Abstract

**Background:**

Performing open carpal tunnel release (oCTR) in an office-based procedure room setting (PR) decreases surgical costs when compared with the operating room (OR). However, it is unclear if the risk of major medical, wound, and iatrogenic complications differ between settings. Our purpose was to compare the risk of major medical complications associated with oCTR between PR and OR settings.

**Methods:**

Utilizing the MarketScan Database, we identified adults undergoing isolated oCTR between 2006 and 2015 performed in PR and OR settings. ICD-9-CM and/or CPT codes were used to identify major medical complications, surgical site complications, and iatrogenic complications within 90 days of oCTR. Multivariable logistic regression was used to compare complication risk between groups.

**Results:**

Of the 2134 PR and 76,216 OR cases, the risk of major medical complications was 0.89% (19/2134) and 1.20% (914/76,216), respectively, with no difference observed in the multivariable analysis (adjusted odds ratio [OR] 0.84; 95% CI 0.53–1.33; *P*=0.45). Risk of surgical site complications was 0.56% (12/2134) and 0.81% (616/76,216) for the PR and OR, respectively, with no difference in the multivariable analysis (OR 0.68; 95% C.I. 0.38–1.22; *P*=0.19). Iatrogenic complications were rarely observed (PR 1/2134 [0.05%], OR 71/76,216 [0.09%]), which precluded multivariable modeling.

**Conclusion:**

These results support a similar safety profile for both the PR and OR surgical settings following oCTR with similar pooled major medical complications, pooled wound/surgical site complications, and iatrogenic complications.

## INTRODUCTION

Carpal tunnel syndrome is the most common compressive neuropathy of the upper extremity. Over 500,000 carpal tunnel releases are performed each year within the United States, with direct costs exceeding 2 billion dollars annually.^1,2^ There has been an increasing emphasis within healthcare to deliver more cost-effective and efficient surgical services while still maintaining the highest quality of patient care.

Substantial literature within the field of hand surgery has suggested that one effective method of surgical cost reduction is the utilization of a procedure room setting (PR), rather than the operating room setting (OR) for minor procedures.^3–13^ Compared with the more traditional OR surgical setting with full sterility utilizing regional or general anesthesia, office-based PR surgical settings for minor hand procedures use field sterility under pure local anesthesia with lidocaine and epinephrine. Generally, a tourniquet is not used in the PR setting. Open carpal tunnel releases (oCTR), trigger finger release, de Quervain release, and other minor procedures are feasible using WALANT techniques (wide-awake, local-only anesthesia, no tourniquet) in the PR setting, which has been proposed to improve the value of care for patients.^3–6,8,9,11,12,14–16^ Specific to oCTR, direct costs may range from four-fold^9^ to 30-fold^3^ greater for the OR, when compared with the PR. In addition to utilizing the WALANT technique, decreased medical consultation and testing with utilization of the PR setting may further lead to lower preoperative and overall costs.^13,14^

Value of care is equivalent to the treatment outcome, or level of improvement, per unit cost.^17–20^ Improving clinical or functional outcomes while maintaining a comparable treatment cost increases the value of care within orthopedic surgery. Additionally, lowering surgical costs while maintaining clinical and functional outcomes also improves the value of care. Utilizing a value-based payment model has expanded efforts to enhance the treatment outcomes and effectiveness of healthcare delivery within the United States and to reduce avoidable costs.^21^

In addition to cost, other vital components in evaluating value of care include clinical and functional outcomes, as well as safety and complication rates. However, there remains a paucity of evidence as to the safety profile of utilizing the PR setting for oCTRs and how it compares to the complication rate of utilizing the OR, which is the traditional setting for CTR surgery using regional, sedation, or general anesthesia. A subjectively low postoperative complication rate has been reported for various hand surgeries performed in the PR setting, although most do not specifically investigate complication profiles or specifically compare complication rates between PR and OR settings.^3,4,6,11,22^ Among studies evaluating PR complications, Leblanc et al published a multicenter noncomparative study evaluating postoperative infection rates following oCTR performed in the PR setting.^10^ Although the results were promising, with rates of 0.4% and 0% for superficial and deep infections, other medical complications and iatrogenic complications to neurovascular or tendon structures were not evaluated. Other studies have attempted to address the PR surgical setting safety by comparing complication rates between PR and OR, reporting zero complications for both settings among oCTR patients and low complication rates for trigger finger release patients.^8,23^ However, both of these studies were limited by small sample sizes and under-powering for the purpose of drawing strong conclusions.^24^

The primary purpose of our study was to evaluate our hypothesis that the risk of major medical complications associated with oCTR was similar between PR and OR settings in a large, geographically-diverse population-based cohort. Our secondary purpose was to evaluate the hypothesis that following oCTR, the risk of surgical site and iatrogenic complications (neurovascular or tendon injury) are similar between PR and OR surgical settings.

## METHODS

### Definition of PR and OR Populations

In this prospective cohort study, individuals aged 18–64 years who underwent oCTR surgery from 7/1/2006 to 6/30/2015 were identified using the IBM MarketScan Commercial Database. Due to the deidentification and limited dataset of the MarketScan database, this study was considered exempt by the University of Utah Institutional Review Board and the Washington University in St. Louis Human Resource Protection Office. Included in this database is information regarding enrollment, medical and outpatient pharmacy claims, data for dependents, employees, and individuals with Consolidated Omnibus Budget Reconciliation Act (COBRA) continuation covered by employer-sponsored and other commercial health insurance plans. The MarketScan database is a prospective database that encompasses over 150 million patients throughout the study’s duration, with information contributed by commercial health insurance plans and employers. Not included in the database are individuals 65 years and older, uninsured individuals, workers’ compensation, government-sponsored plans, and individuals with other types of private insurance.

Using the current procedural terminology, fourth edition (CPT-4) code 64721 for oCTR coded by a provider, we identified individuals undergoing oCTR from the inpatient and outpatient medical claims files. A diagnosis of carpal tunnel syndrome using the International Classification of Disease, ninth revision, clinical modification 354.0 (ICD-9-CM 354.0) on the claim line for the procedure was required. To assess complications and comorbidities, 180 days of medical insurance coverage enrollment before surgery and 90 days following surgery was required. The OR surgical setting was identified by utilizing uniform billing codes to identify if the procedure took place in the OR based on the revenue center code for major OR services (0360, 0361). The PR surgical setting was identified on the surgeon claim line via a place-of-service code of 11 (in-office procedure). Additionally, there was no associated OR revenue center code for OR/ambulatory surgery services (0360, 0361, or 0490). There was also no associated claim for general, regional, sedation, or nerve block anesthesia on the day of the surgery.

Persons with unrelated procedures on the date of oCTR were excluded to compare outcomes in patients with isolated oCTR depending on the surgical setting. Unrelated procedures comprised a broad range of CPT-4 codes with the exception of iatrogenic-related procedures and nerve block codes. To avoid excluding patients with intraoperative complications that were treated during the index oCTR surgery, we included procedures on the oCTR date that were potentially related to address possible iatrogenic injuries.

The exclusions for our study are summarized in Supplemental Digital Content 1. (**See appendix, Supplemental Digital Content 1,** which displays Appendix I: Summary of study exclusions. Appendix II: Coding used to identify and exclude noniatrogenic injuries. Appendix III: Coding used to identify major medical complications. Appendix IV: Coding used to identify surgical wound complications. Appendix V: Coding used to identify iatrogenic surgical complications. Appendix VI: Summary and comparison of comorbidities for PR and OR groups. http://links.lww.com/PRSGO/B707.)

We included only the initial procedure that met qualification requirements for patients who had multiple oCTR procedures during the study interval. We excluded individuals with noniatrogenic injuries (eg, rupture, injury to the nerves/vessels/tendons associated with oCTR, open wound) (**SDC1: Appendix II,** **http://links.lww.com/PRSGO/B707**) that occurred within 30 days before surgery. In addition, oCTR procedures in persons coded for a related surgery in the 180 days before the oCTR date, suggesting that the oCTR was secondary to the prior procedure, were excluded. To focus on nonemergent, uncomplicated oCTR procedures, we excluded any procedures associated with an emergency department visit or during an inpatient admission.

### Identification of Underlying Comorbidities and Other Potential Risk Factors for Complications

We utilized the Elixhauser classification to identify comorbidities.^25,26^ Facility coding in one or more inpatient hospitalization and/or 2 or more outpatient claims spaced at least 30 days apart were required, excluding outpatient claims regarding alcohol abuse, tobacco abuse, drug abuse, weight loss, or obesity.^27^ Active smoking status was identified using diagnostic coding (ICD-9 305.1, 649.0x).

### Identification of Major Medical and Surgical Outcomes

The primary outcome of our study was any major medical complications within 90 days following oCTR. All medical complications of interest were acute events only. Thus, a single code was necessary within 90 days of the surgery. We determined major medical complications of interest as ICD-9-CM diagnosis/procedure codes for any of the following: respiratory failure, cardiac/respiratory arrest, congestive heart failure exacerbation, acute myocardial infarction, acute deep vein thrombosis, acute pulmonary embolism (PE), acute renal failure, postoperative shock, acute stroke, transient ischemic attack, and death (specific coding provided in **SDC1: Appendix III.** **http://links.lww.com/PRSGO/B707**).

We utilized ICD-9-CM diagnosis or CPT-4 codes for surgical site complications. Such complications included any nonhealing wound, wound disruption, surgical site infection, hemorrhage complicating a procedure, hematoma, and seroma (specific coding provided in **SDC1: Appendix IV.** **http://links.lww.com/PRSGO/B707**). We defined iatrogenic complications as any new neurovascular or tendon structure injury that was not previously coded within the 6 months pre-oCTR but was diagnosed and/or surgically-treated within 90 days post-oCTR (specific coding provided in **SDC1: Appendix V**. **http://links.lww.com/PRSGO/B707**).

### Statistical Methods

To determine factors associated with medical, surgical, and iatrogenic complications, we utilized a multivariable logistic regression. We used the location of the surgery as the primary exposure forced in the model. Variance inflation factors were utilized to assess the potential multicollinearity of independent variables. In the initial full models, we included variables with a *P* value less than 0.2 in bivariate analysis or with clinical/biologic plausibility. We defined a *P* value less than 0.1 as the threshold for retention of all variables as they were removed in a stepwise backward manner. We compared the demographics and complication rates between PR and OR groups using Fisher’s exact test or chi-squared tests for binary variables and Student’s *t* or Mann-Whitney U tests for continuous variables.

A power calculation was performed utilizing the observed ratio of OR to PR cases (35.7:1). To achieve 80% power at a 95% confidence level, a total of 1488 PR patients and 53,122 OR patients would be needed to discriminate a difference of 0.75% for major medical complications (0.75% versus 1.5%) on the two-tailed two-proportion test. All statistical analyses were performed in SAS version 9.4 (Cary, N.C.), with *P* < 0.05 considered statistically significant.

## RESULTS

We identified 2,134 patients treated with isolated oCTR in the PR, and 76,216 in the OR. Subjects were excluded under the following conditions: lack of health insurance coverage in the 180 days before index and/or the 90 days post procedure (n=83,131), presence of coding for a noniatrogenic injury in the 30 days before oCTR (n=2835), a related surgery in the 180 days before the oCTR (n=8526), another CPT code in the surgical range on the oCTR date (n=79,699), evidence of an ER visit on the oCTR date (n=2914), oCTR performed after the date of admission in the inpatient setting (n=49), inability to determine performance in the OR or PR (n=114,606), and subsequent oCTR procedures (n=16,441). Of included subjects, the mean age was 50 ± 9 years, with 66.8% being women. Additional demographics are provided in Table 1. Smoking status and the frequency of comorbidities for PR and OR groups are shown in SDC1. (**See SDC 1: Appendix VI.** **http://links.lww.com/PRSGO/B707**.)

**Table 1.** Demographic Data

Variable	PR (n=2134)	OR (n=76,216)	*P*
Age			
18–39	258 (12.09%)	10,992 (14.42%)	Reference
40–49	538 (25.21%)	19,819 (26.00%)	<0.001
50–59	911 (42.69%)	32,507 (42.65%)	<0.01
60 and older	427 (20.01%)	12,898 (16.92%)	<0.001
Anesthesia type			
General or regional	0 (0.00%)	63,351 (83.12%)	NA
Sedation	0 (0.00%)	100 (0.13%)	—
Local	2134 (100%)	12,765 (16.75%)	—
Postoperative nerve block	0 (0.00%)	2405 (3.16%)	—
Insurance type			
HMO or POS with capitation	849 (39.78%)	8889 (11.66%)	<0.001
All other plan types	1285 (60.22%)	67,327 (88.34%)	—
Region			
Northeast	239 (11.20%)	12,433 (16.31%)	0.651
North Central	491 (23.01%)	27,807 (36.48%)	0.069
South	519 (24.32%)	28,068 (36.83%)	Reference
West	885 (41.47%)	7908 (10.38%)	0.006
Residence type			
Urban	1536 (71.98%)	55,198 (72.42%)	0.65
Rural	598 (28.02%)	21,018 (27.58%)	—
Gender			
Men	717 (33.60%)	25,275 (33.16%)	0.672
Women	1417 (66.40%)	50,941 (66.84%)	—

Continuous variables were analyzed using logistic regression, and categorical variables were analyzed using the chi-squared test.

### Major Medical Outcomes

**Fig. 1.**
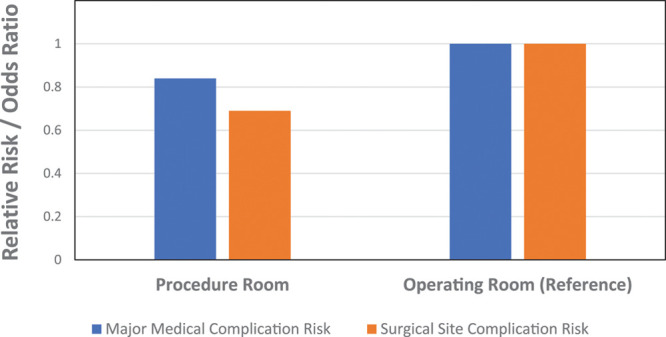
Illustration of relative risks/odds ratios for pooled major medical complications and surgical site complications between procedure room and operating room settings. Note that there is no statistical difference between adjusted pooled major medical and adjusted pooled surgical site complication risks between procedure room and operating room settings.

The crude pooled risk of a major medical complication was 0.89% (19/2134) for the PR group and 1.20% (914/76,216) for the OR group (*P*=0.19; Table 2). There was no significant difference in major medical complication risk based on the surgical setting in the multivariable analysis (adjusted odds ratio 0.84 for PR versus OR; 95% CI 0.53–1.32; *P*=0.45). This is illustrated in Figure 1. In contrast, hypertension, diabetes, drug abuse, anemia, rheumatoid arthritis/collagen vascular disease, chronic pulmonary disease, neurologic disorders, psychological disorders/psychoses, hypothyroidism, older age, and male gender were associated with a significantly increased risk of a major medical complication (Table 3).

**Table 2.** Unadjusted Risk of Major Medical Complications

	PR (n=2134)	OR (n=76,216)	P*
Pooled major medical complications	19 (0.89%)	914 (1.20%)	0.194
Acute MI	2 (0.09%)	47 (0.06%)	0.387
Acute stroke	12 (0.56%)	436 (0.57%)	0.953
TIA	4 (0.19%)	122 (0.16%)	0.589
Death	0 (0.00%)	4 (0.01%)	>0.999
Cardiac/respiratory arrest	1 (0.05%)	10 (0.01%)	0.262
Respiratory failure	1 (0.05%)	67 (0.09%)	>0.999
Acute PE	0 (0.00%)	96 (0.13%)	0.118
Acute DVT	2 (0.09%)	128 (0.17%)	0.590
Congestive heart failure exacerbation	0 (0.00%)	30 (0.04%)	>0.999
Acute renal failure	2 (0.09%)	130 (0.17%)	0.591
Postoperative shock	0 (0.00%)	1 (0.00%)	>0.999

*Comparisons for pooled major medical complications, and acute stroke, were determined using chi-squared test. The other comparisons were made using Fisher’s exact test.

**Table 3.** Multivariable Logistic Regression Model for Pooled Major Medical Complication Risk

Variable*†	Coefficient	95% Wald Confidence Limits		*P*
		Upper Limit	Lower Limit	
Surgical setting (PR versus OR)	0.84	0.53	1.33	0.450
Elixhauser comorbidity index variables	—	—	—	—
Anemia	3.33	2.46	4.51	<0.0001
Rheumatoid arthritis/collagen vascular disease	2.21	1.62	3.02	<0.0001
Chronic pulmonary disease	2.39	1.93	2.98	<0.0001
Diabetes	2.16	1.85	2.52	<0.0001
Drug abuse	2.08	1.19	3.64	0.010
Hypertension	1.80	1.56	2.08	<0.0001
Neurologic disorders	2.16	1.46	3.19	0.000
Psychological disorders/psychosis	1.42	1.05	1.93	0.024
Hypothyroidism	1.46	1.10	1.92	0.008
Sex (men versus women)	1.42	1.24	1.62	<0.0001
Age category (versus 18–39)	—	—	—	—
40–49	1.95	1.36	2.81	<0.0001
50–59	3.25	2.32	4.56	<0.0001
60+	4.81	3.40	6.81	<0.0001

*Note that the following additional variables were included in the model but were eliminated through a backward term selection method: alcohol abuse, obesity, solid tumor without metastasis, and smoking. Rural (versus urban) residence, region, and insurance type were also nonsignificant.

†Note that the following Elixhauser comorbidity variables were not analyzed in this model due to insignificance in univariate analysis (*P* > 0.20) or due to counts < 5: AIDS, chronic blood loss anemia, congestive heart failure, coagulopathy, depression, liver disease, lymphoma, fluid and electrolyte disorders, metastatic cancer, paralysis, peripheral vascular disease, pulmonary circulation disease, renal failure, valvular disease, and weight loss.

### Surgical Outcomes

The crude pooled risk of surgical site complications was 0.56% (12/2134) for the PR group and 0.81% (616/76,216) for the OR group (*P*=0.21). The risk of surgical site complications for both settings are provided in Table 4. In the multivariable analysis, no significant association existed between surgical setting and surgical site complications (adjusted odds ratio 0.68 for PR versus OR; 95% C.I. 0.38–1.22; *P*=0.19; Table 5). This is illustrated in Figure 1. Factors associated with an increased risk of surgical site complications included chronic pulmonary disease, diabetes, obesity, psychological disorders/psychoses, solid tumor, male gender, and smoking, while the north central region of the United States (compared with the South) was associated with a significantly lower risk of surgical site complications (Table 5).

**Table 4.** Unadjusted Risk of Surgical Site Complications

	PR (n=2134)	OR (n=76,216)	P*
Pooled surgical site complications	12 (0.56%)	616 (0.81%)	0.209
Surgical site infection	10 (0.47%)	303 (0.40%)	0.608
Surgical site wound disruption	1 (0.05%)	235 (0.31%)	0.024
Surgical site seroma	0 (0.00%)	28 (0.04%)	>0.999
Surgical site hematoma	1 (0.05%)	25 (0.03%)	0.512
Surgical site nonhealing wound	1 (0.05%)	87 (0.11%)	0.735
Hemorrhage complicating a procedure	0 (0.00%)	24 (0.03%)	>0.999

*Comparisons for pooled surgical site complications, and surgical site infection, were determined using Chi-squared test. The other comparisons were made using Fisher’s Exact Test.

**Table 5.** Multivariable Logistic Regression Model for Pooled Surgical Site Complication Risk

Variable*†	Coefficient	95% Wald Confidence Limits		*P*
		Upper Limit	Lower Limit	
Surgical setting (PR versus OR)	0.69	0.38	1.22	0.200
Elixhauser comorbidity index variables	—	—	—	—
Chronic pulmonary disease	1.67	1.21	2.30	0.002
Diabetes	1.64	1.33	2.03	<0.0001
Obesity	1.53	1.23	1.89	0.000
Psychological disorders/psychosis	1.64	1.15	2.33	0.007
Solid tumor	2.60	1.57	4.30	0.000
Region (versus South)	—	—	—	—
North central	0.82	0.68	0.98	0.032
North east	0.85	0.67	1.08	0.173
West	1.17	0.92	1.51	0.205
Gender (men versus women)	1.32	1.13	1.56	0.001
Smoking	1.36	1.10	1.69	0.004

*Note that the following additional variables were included in the model but were eliminated through a backward term selection method: age, depression, hypertension, hypothyroidism, and neurologic disorders.

†Note that the following Elixhauser comorbidity variables were not analyzed in this model due to insignificance in univariate analysis (*P* > 0.20) or due to counts < 5: AIDS, chronic blood loss anemia, congestive heart failure, coagulopathy, depression, liver disease, lymphoma, fluid and electrolyte disorders, metastatic cancer, paralysis, peripheral vascular disease, pulmonary circulation disease, renal failure, valvular disease, and weight loss. Rural (versus urban) residence and insurance type were also nonsignificant.

The crude pooled risk of iatrogenic surgical complications was 0.05% (1/2134) for the PR group, and 0.09% (71/76,216) for the OR group (*P* > 0.99 Fisher’s exact test; Table 6). Multivariable modeling was precluded due to the lack of adequate sample size secondary to the rarity of iatrogenic surgical complications.

**Table 6.** Unadjusted Rates of Iatrogenic Surgical Complication Risk

	PR (n=2060)	OR (n=73925)	P*
Pooled iatrogenic complications	1 (0.05%)	71 (0.09%)	>0.999
New nerve injury	1 (0.05%)	41 (0.05%)	>0.999
New blood vessel injury	0 (0.00%)	8 (0.01%)	>0.999
New tendon injury	0 (0.00%)	22 (0.03%)	>0.999
Iatrogenic injury	1 (0.05%)	30 (0.04%)	0.575

*Comparisons were made using Fisher’s exact test.

## DISCUSSION

Our main finding was that oCTR performed in the PR setting was associated with a low risk of pooled major medical complications that was similar to the rate observed for patients treated in a traditional OR setting. Within 90 days of surgery, we observed that 0.89% of PR patients and 1.20% of OR patients suffered a major medical complication, which was not significantly different. In light of how this study was powered, we conclude that the risk of major medical complications for PR and OR settings are no different within a threshold of less than 0.75%. These findings suggest that performing oCTR in the PR setting is safe and comparable to choosing the OR as the surgical setting from a medical safety standpoint.

Although we could not identify a large comparative study comparing complication rates between PR and OR settings, our results in regard to major medical complications following oCTR are consistent with previous literature for minor hand surgeries. Lipira et al found the risk of myocardial infarction, pulmonary embolism, shock, stroke, hemorrhage, or nerve injury for outpatient hand surgery to each be less than 0.1%.^24^ Although an OR comparison group was not studied, Bismil et al additionally found no intraoperative complications in their analysis of 1000 consecutive cases of various upper limb orthopedic surgeries utilizing a safe, efficient, and effective one-stop (patient seen and treated in one appointment) wide-awake (local anesthesia only) hand surgery service they developed as an alternative to the OR surgical setting.^28^

Secondary study findings pertain to the risk of pooled surgical site and wound complications, which were infrequent and similar between PR and OR settings. Our observed risk of wound complications, ranging from 0.56% in the PR group to 0.81% for the OR, is consistent with the 0.32% infection rate observed by Werner et al among over 450,000 CTR surgeries performed.^29^ A breakdown by surgery setting or anesthesia type was not provided, and the authors excluded patients undergoing simultaneous distal radius ORIF, but did not exclude other concomitant procedures. Our study also found a 0.56% surgical site infection rate for the PR group and 0.81% for the OR group, consistent with Lipira et al’s reported 1.1% in 10,646 patients who underwent surgical procedures of the hand or wrist^24^ and Tosti et al’s 0.66% after 600 consecutive elective soft tissue hand surgeries.^30^ Maliha et al found no difference in intraoperative and postoperative complication rates, infection rates, wound healing complications, or recurrences for patients who underwent trigger finger release in an OR versus PR settings.^8^ However, this study was limited in statistical power, with only 39 PR and 37 OR patients, which precludes forming statistically-sound, firm conclusions about potential differences in these rare complications. It is important to note that although we found a significantly lower rate of wound disruption in the PR group (0.05% versus 0.31%), this unadjusted finding did not account for the higher rate of comorbidities in the OR group, including diabetes and obesity, and in terms of the main secondary outcome (pooled complication risk), there was no difference between PR and OR groups.

Lastly, we found low rates of pooled iatrogenic complications in our cohorts. Specifically, the pooled iatrogenic complication rate was 0.09% for the OR group and 0.05% for the PR group, with no difference between surgical settings. Due to the rarity of these complications, we cannot draw strong conclusions, other than the observation that these issues are infrequent in general following oCTR in both surgical settings. This finding is congruent with prior literature that observed no iatrogenic complications among 1404 procedure room surgical encounters^31^ and absence of such complications in two independent smaller studies.^8,28^ This finding is additionally supported by Lipira et al, who detailed a nerve injury rate of less than 0.1% in both surgical settings for their cohort of 10,646 patients who had undergone surgical procedures of the hand or wrist in the inpatient or outpatient setting.^24^

Our study contains several limitations that deserve mention. Our power calculation utilized the observed ratio of OR to PR cases (35.7:1) to achieve 80% power at a 95% confidence level, which allowed us to calculate the number of patients needed in each surgical setting to discriminate a difference of less than 0.75% for major medical complications on the two-tailed two-proportion test. We felt that we powered our study to a clinically meaningful low threshold, but the determination of the most appropriate threshold is somewhat subjective. Our study had potential for coding errors given the administrative database that was utilized to identify our cohort. The MarketScan database is limited in scope and is not comprehensive of all data variables. Due to the claims nature of the database, it is possible that we are under-capturing minor complications that are not tied to reimbursement. However, major complications that require re-operation and are thus tied to reimbursement are likely to be coded more accurately. We did not collect data regarding local anesthesia complications because we were unable to achieve this level of granularity with this database study, although this has been noted to be very rare or not reported in large series of WALANT cases.^10,32^ The generalizability of our study to older or more socially deprived patient populations is unclear, given our utilization of the MarketScan database, which solely comprises commercially insured patients younger than 65 years of age. Our study only looked at complications within a 90-day postoperative period. However, the NSQIP database^33^ only looks at 30 days postoperatively for complications; so we feel our 90-day period is fairly comprehensive, but it is possible that later complications or revisions could be missed by our analysis. We also excluded a large number of patients. It is possible that these exclusions could have an impact on the results, but we do not know if those excluded differ from those included in our study. The interpretation of our results is somewhat limited in regard to commenting on what extent the surgical setting versus the anesthesia type impacted the observed similar safety profile and complications among the two patient groups, as the PR setting is linked to local-only anesthesia. Lastly, the rarity of iatrogenic surgical complications precluded our ability to perform a multivariable analysis to control for demographics and comorbidities among cohorts.

## CONCLUSIONS

In conclusion, our findings support a similar safety profile for OR and PR surgical settings for patients who underwent oCTR. Rates of pooled major medical complications were low and similar following oCTR performed in either setting, as were pooled surgical site/wound complications and iatrogenic complications. In light of these findings supporting a similar safety profile, prior studies illustrating a substantial cost reduction associated with use of the PR setting for oCTR when compared with the OR,^3–13^ and similar clinical outcomes following oCTR performed in PR and OR settings,^33^ we conclude that the value of utilizing the PR setting for oCTR is greater than that of the OR.

## Supplementary Material


