# Supplementary material for: Comparison of Complication Risk for Open Carpal Tunnel Release: In-office versus Operating Room Settings
Source: Plast Reconstr Surg Glob Open. 2021 Jul 12;9(7):e3685. doi: 10.1097/GOX.0000000000003685 (PMC8274797; doi:10.1097/GOX.0000000000003685)
Supplement: Supplementary file 1 [file gox-9-e3685-s001.pdf]

Appendix I – **Summary of study exclusions**

Exclusion numbers:

- 386,551 open CT releases between 7/1/2006-6/30/2015 in persons aged 18-64 years with residence in the U.S.
    - Dropped 83,131 procedures due to lack of health insurance coverage in the 180 days prior to and/or 90 days post-surgery
    - 2,835 dropped due to diagnosis codes for non-iatrogenic injuries in the 30 days prior to or on the date of oCTR
    - 8,526 dropped due to CPT codes for wrist/hand surgeries in the 180 days prior to or on the oCTR date
    - 79,699 dropped due to another surgical/procedure CPT code on the oCTR date
    - Dropped 2,914 surgeries due to evidence of ER visit on the oCTR date
    - Dropped 49 surgeries due to surgery after the date of admission during an inpatient hospitalization
    - Dropped 114,606 surgeries when performance of the procedure in the OR or PR could not be determined
    - Dropped 16,441 when limiting to first eligible oCTR per patient

Appendix II – **Coding used to identify and exclude non-iatrogenic injuries**

- All CPT codes in the range of 10021-69990, EXCEPT the following codes for iatrogenic injuries and nerve block:
    - Iatrogenic injuries: 64856, 64857, 35206, 35207, 37618, 26356, 26350
    - Nerve block: 64415, 64416, 64417, 64450

Appendix III – Coding used to identify major medical complications

Acute MI - 410.01, 410.11, 410.21, 410.31, 410.41, 410.51, 410.61, 410.71, 410.81, 410.91

Acute Stroke –

- Subarachnoid: 430
- Intracerebral hemorrhage: 431
- Other: 432.0 (nontraumatic extradural), 432.1 (subdural), 432.9 (unspecified ICH)
- Occlusion of precerebral arteries: 433.00/.01/.10/.11/.20/.21/.30/.31/.80/.81/.90/.91
- Occlusion of cerebral arteries: 434.00/.01/.10/.11/.90/.91
- Acute but ill-defined: 436

TIA - 435.0/.1/.2/.3/.8/.9

Death - 798.1/.2/.9, (Discharge status was also used to identify death)

Cardiac/Respiratory Arrest -

- Cardiac arrest: 427.5
- Respiratory arrest: 799.1
- Due to a procedure: 977.1

Respiratory Failure

- Failure: 518.81
- Failure secondary to surgery: 518.51
- Acute on chronic: 518.84
- ICD9 procedure code: 96.72

Acute PE - PE: 415.11/.13/.19

Acute DVT –

- Acute LE DVT: 453.40/.41/.42
- Acute DVT of other veins: 453.81-89

Congestive Heart Failure Exacerbation - 428.21, 428.23, 428.31, 428.33, 428.41, 428.43
Acute Renal Failure - 584.5, 584.6, 584.7, 584.8, 584.9
Post-Operative Shock - 998.00, 998.01, 998.02, 998.09

Appendix IV – Coding used to identify surgical wound complications
SSI – 998.5
Surgical Site Wound - 998.30, 998.31, 998.32, 998.33
Surgical Site Seroma – 998.13
Surgical Site Hematoma – 998.12
Surgical Site Non-Healing Wound – 998.83
Hemorrhage complicating a procedure – 998.11

Appendix V – Coding used to identify iatrogenic surgical complications
**Note: Nerve injuries, blood vessel injuries, or tendon injuries are only counted post-surgery if the patient does not have the same injury coded in the 90 days prior to index. Injuries coded on the surgery date will be counted as post-index. Also only identified procedures on provider non-assistant claims.
New Nerve Injury

- Diagnosis codes: 955.1, 955.2, 955.3, 955.4, 955.5, 955.6, 955.7, 955.8, 955.9
- CPT codes: 64856, 64857

New Blood Vessel Injury

- Diagnosis codes : 903.2, 903.3, 903.4, 903.5, 903.8, 903.9, 997.79
- CPT codes: 35206, 35207

New Tendon Injury

- Diagnosis codes: 998.2
- CPT codes: 26350, 26356

Iatrogenic Injury

- Diagnosis codes: 998.2, E870-E876
- CPT codes: 64856, 64857, 35206, 35207, 37618, 26356, 26350

Appendix VI - Summary and Comparison of Comorbidities for PR and OR Groups

Comorbidities	OR Procedures (n=76,216)	PR Procedures (n=2,134)	p-value
**Active smoking**	9,521 (12.49%)	95 (4.45%)	<.01
**Elixhauser Comorbidities**			
AIDS	39 (0.05%)	0 (0.00%)	0.63
Alcohol abuse	440 (0.58%)	18 (0.84%)	0.11
Deficiency anemias	798 (1.05%)	13 (0.61%)	0.05
Rheumatoid arthritis/collagen vascular disease	1,371 (1.80%)	63 (2.95%)	<.01
Chronic blood loss anemia	44 (0.06%)	0 (0.00%)	0.63
Congestive heart failure	207 (0.27%)	6 (0.28%)	0.93
Chronic pulmonary disease	2,749 (3.61%)	46 (2.16%)	0.00
Coagulopathy	107 (0.14%)	1 (0.05%)	0.38
Depression	2,527 (3.32%)	52 (2.44%)	0.03
Diabetes (uncomplicated or complicated)	7,976 (10.46%)	155 (7.26%)	<.01
Drug abuse	459 (0.60%)	21 (0.98%)	0.03
Hypertension (uncomplicated or complicated)	14,283 (18.74%)	190 (8.90%)	<.01
Hypothyroidism	2,502 (3.28%)	30 (1.41%)	<.01
Liver disease	202 (0.27%)	6 (0.28%)	0.89
Lymphoma	88 (0.12%)	3 (0.14%)	0.74
Fluid and electrolyte disorders	391 (0.51%)	11 (0.52%)	0.99
Metastatic cancer	51 (0.07%)	1 (0.05%)	1.00
Other neurological disorders	765 (1.00%)	8 (0.37%)	0.00
Obesity	8,068 (10.59%)	153 (7.17%)	<.01
Paralysis	79 (0.10%)	5 (0.23%)	0.08
Peripheral vascular disease	213 (0.28%)	5 (0.23%)	0.70
Psychological disorders/psychoses	2,399 (3.15%)	70 (3.28%)	0.73
Pulmonary circulation disease	98 (0.13%)	1 (0.05%)	0.53
Chronic kidney disease	314 (0.41%)	5 (0.23%)	0.20
Solid tumor without metastasis	780 (1.02%)	16 (0.75%)	0.21
Valvular disease	231 (0.30%)	2 (0.09%)	0.10
Weight loss	320 (0.42%)	5 (0.23%)	0.19
